# Risk factors analysis of acute kidney injury following open thoracic aortic surgery in the patients with or without acute aortic syndrome: a retrospective study

**DOI:** 10.1186/s13019-020-01257-1

**Published:** 2020-08-07

**Authors:** Xiaochun Ma, Jinzhang Li, Yan Yun, Diming Zhao, Shanghao Chen, Huibo Ma, Zhengjun Wang, Haizhou Zhang, Chengwei Zou, Yuqi Cui

**Affiliations:** 1 Department of Cardiovascular Surgery, Shandong Provincial Hospital affiliated to Shandong First Medical University, No.324 Jingwu Road, Jinan, 250021 Shandong China; 2 Department of Cardiovascular Surgery, Shandong Provincial Hospital affiliated to Shandong University, No.324 Jingwu Road, Jinan, 250021 Shandong China; 3 College of Basic Medicine, Capital Medical University, Beijing, China; 4 Department of Radiology, Qilu Hospital of Shandong University, No.107 West Wenhua Road, Jinan, 250012 Shandong China; 5 Qingdao University Medical College, Qingdao University, 308 Ningxia Road, Qingdao, 266071 Shandong China; 6 Department of Cardiology, Shandong Provincial Hospital affiliated to Shandong First Medical University, No.324 Jingwu Road, Jinan, 250021 Shandong China; 7 Center for Precision Medicine and Division of Cardiovascular Medicine, University of Missouri School of Medicine, Columbia, MO 65212 USA

**Keywords:** Acute aortic syndrome, Acute kidney injury, Independent predictor, Open thoracic aortic surgery

## Abstract

**Background:**

The acute kidney injury (AKI) remains a frequent complication following open thoracic aortic surgery (OTAS) and worsens the postoperative prognosis. It remains unclear that whether the predictors of AKI following OTAS are different in the patients with or without acute aortic syndrome (AAS).

**Methods:**

Preoperative and intraoperative variables were compared between the patients with or without AKI, and were further analyzed for identifying the potential predictors of postoperative AKI. Subgroup analysis was conducted in the patients with or without AAS, respectively.

**Results:**

AKI after OTAS occurred in 57.6% of the overall cohort, 70.1% of the patients with AAS and 46.7% of the patients without AAS. In the multivariate analysis, history of hypertension (OR 1.011, 95% CI: [1.001–1.022], *p* = 0.04), preoperative platelet (OR 0.995, 95% CI: [0.991–0.999], *p* = 0.006) and operation time (OR 1.572, 95% CI: [1.355–1.823], *p* < 0.001) were identified as independent predictors of postoperative AKI for the overall cohort; CPB time (OR 1.020, 95% CI: [1.009–1.031], *p <* 0.001) and preoperative LMR (OR 0.823, 95% CI: [0.701–0.966], *p* = 0.02) as independent predictors for the patients with AAS; age (OR 1.045, 95% CI: [1.015–1.076], *p* = 0.003), preoperative platelet (OR 0.993, 95% CI: [0.988–0.998], *p* = 0.04) and operation time (OR 1.496, 95% CI: [1.166–1.918], *p* = 0.002) as independent predictors for the patients without AAS.

**Conclusions:**

The patients with AAS carry a higher risk for postoperative AKI compared with those without AAS. The predictive factors for postoperative AKI after OTAS are different for AAS- and non-AAS subgroups and operation time, CPB time and preoperative platelet are modifiable predictors of AKI.

## Introduction

The AKI complicates 10–70% patients undergoing OTAS, depending on the distinct definitions of AKI [1–7]. This complication predicts a dismal prognosis and increases the postoperative mortality and morbidity. Despite the recent advancements in cardiopulmonary bypass (CPB), anesthesia and intensive care, the incidence of AKI following OTAS remains high [8]. The AKI contemporarily represents a huge challenge for cardiovascular surgeons as no definite therapy has thus been developed. Though renal replacement therapy (RRT) is a feasible therapeutic option, modifying those potential risk factors could fundamentally prevent the occurrence of AKI and improves the short- and long-term outcomes [2, 9–12].

The primary objective of this retrospective cohort study was to clarify that whether different risk factors predict AKI after OTAS in the patients with or without AAS.

## Materials and methods

### Study design and participants

**Fig. 1:**
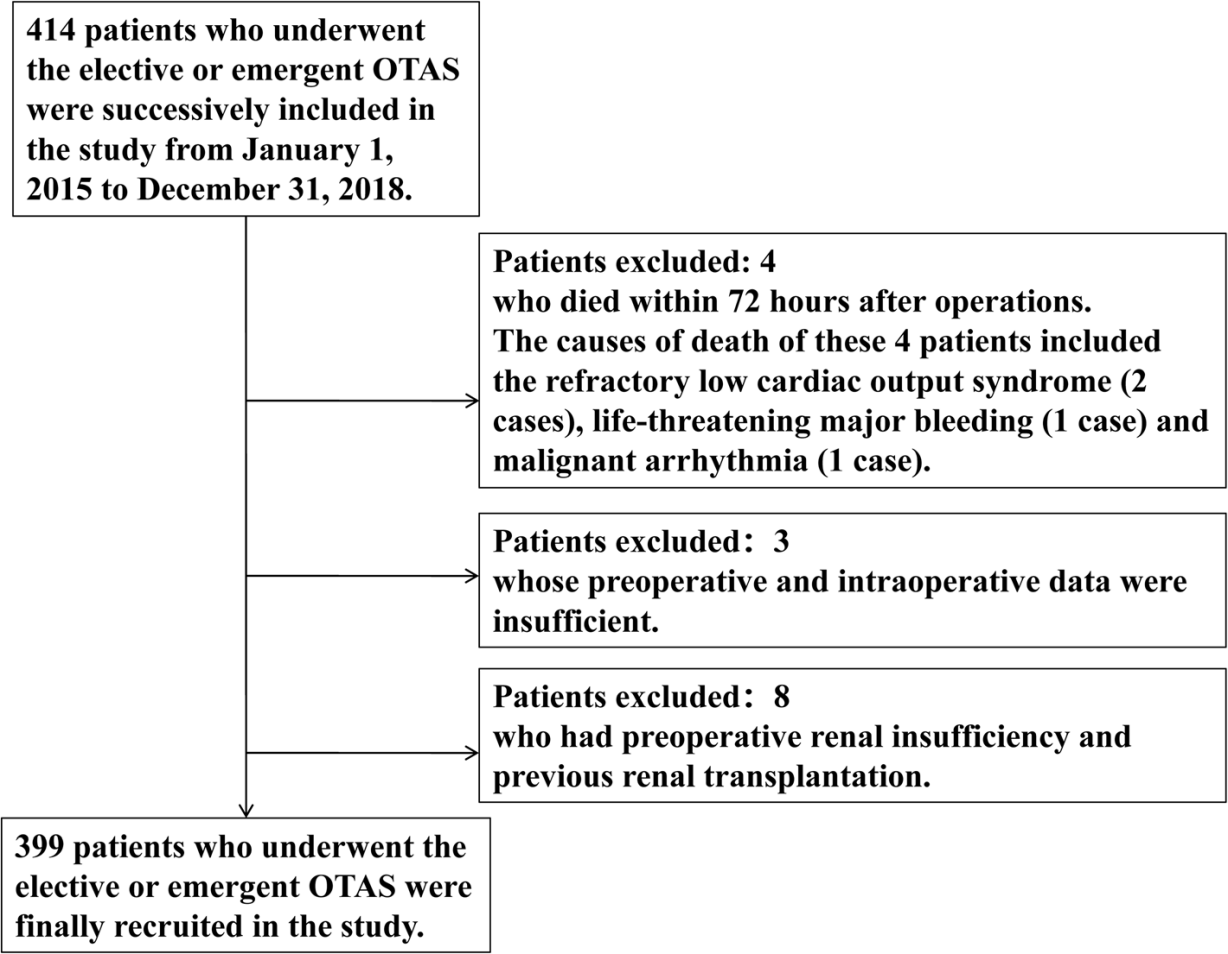
Flow chart of participants inclusion and exclusion. Figure 1 depicts the process of participants inclusion and exclusion

This single-center retrospective cohort study was approved by the Ethics Committee of Shandong Provincial Hospital and the written informed consent was waived due to the retrospective design. 399 consecutive patients were eventually recruited who underwent the elective or emergent OTAS between January 1, 2015 to December 31, 2018. The patients were excluded from the cohort who died within 72 h after operations or whose preoperative and intraoperative data were insufficient or who had preoperative renal insufficiency or previous renal transplantation (Fig. 1). The study was conducted following the Good Clinical Practice (GCP) as well as principles of the Declaration of Helsinki. The clinical data of those patients were carefully collected from admission to discharge. All the operations were completed by an identical surgical team. In this study, AAS represents a series of acute and catastrophic aortic lesions including acute aortic dissection, intramural hematoma, penetrating aortic ulcer, and rupture of aorta due to aortic aneurysm or trauma. Non-AAS represents the non-acute and non-catastrophic thoracic aortic lesions that require elective surgical treatment. The included 399 patients were divided into the AAS subgroup and non-AAS subgroup for further analysis. Potential demographic or perioperative variables considered associated with postoperative AKI were included on the basis of clinical experiences and literature review.

### Definition of AKI following OTAS and postoperative outcomes

The primary outcome of this cohort study was AKI after OTAS. The diagnosis of AKI was established according to the modified Kidney Disease: Improving Global Outcomes guidelines (KDIGO) [13]. The postoperative AKI was defined as an increase of > 50% of the baseline within the first 7 days or > 0.3 mg/dL (26.5 umol/L) within the first 48 h in the postoperative serum creatinine (Scr). The baseline Scr was ascertained based on the latest preoperative tests. And the AKI was further graded as stage I, II and III according to the KDIGO classification and the patients were categorized in line with their highest levels of postoperative Scr. Besides, the postoperative outcomes attributable to AKI included RRT, length of ICU stay and ICU stay > 7 days, intubation time and time > 5 days, stroke, redo surgery and in-hospital mortality.

### Surgical details

The Surgical details were summarized in the supplementary material.

### Statistical analysis

Statistical analysis was performed using SPSS Statistics 25.0. Categorical variables were expressed as frequencies (n) with percentages (%). Continuous variables were expressed as mean ± standard deviation (SD) or median (quartile), depending on the data dispersion. The t-test was used if a continuous variable conformed normal distribution. And non-parametric Mann-Whitney U test was applied if a continuous variable was consistent with skewed distribution. A chi-squared or a Fisher’s exact test was used for categorical variables when necessary. The univariate and multivariate analyses were performed using binary logistic regression analysis. The candidate univariates (with a p valve less than 0.1) as well as those possible predictive variables were included in the multivariate predictive model. And the predictive model was further evaluated using the receiver operating curve (ROC) and the Hosmer-Lemeshow test. Except that the overall cohort were analyzed as above-mentioned, the subgroup analyses were additionally conducted for the patients with or without AAS. All the statistical tests were two-sided and a p valve less than 0.05 was considered statistically significant.

## Results

### Patient characteristics

A total of 399 patients were enrolled in this retrospective cohort study and the patient characteristics of overall cohort, AAS subgroup and non-AAS subgroup were shown respectively in the supplementary material. The patients were excluded who died within 72 h after operations (*n* = 4), whose preoperative and intraoperative data were insufficient (*n* = 3) as well as who had preoperative renal insufficiency and previous renal transplantation (*n* = 8). The causes of death of these 4 patients included the refractory low cardiac output syndrome (*n* = 2), life-threatening major bleeding (*n* = 1) and malignant arrhythmia (*n =* 1). The surgical options of overall cohort were presented in the supplementary Table 1. And the surgical options of AAS subgroup and non-AAS subgroup were showed in supplementary Table 2 and supplementary Table 3, respectively.

### Incidence of AKI after open thoracic aortic surgery

#### Postoperative AKI in the overall cohort

According to the modified KDIGO’s Criteria, the incidence of AKI after aortic operation was 57.6% (230 in 399), among whom 153 patients (38.3%) met the criteria of stage 1, 49 patients (12.3%) satisfied the criteria of stage 2, and 28 patients (7.0%) reached the stage 3 (Table 1).

**Table 1:** Characteristics of the Overall Cohort

	No AKI	AKI			*P*1 Value^*^	*P*2 Value^#^
		Stage 1	Stage 2	Stage 3		
**Patient population (n)**	169	153	49	28		
**Demographic data**						
Age (y)	52.0 (18.0)	54.0 (16.5)	50.0 (13.0)	41.5 (17.5)	0.9	0.004
Sex, male (n)	112 (66.3%)	116 (75.8%)	34 (69.4%)	21 (75.0%)	0.08	0.3
Height (cm)	170.0 (13.0)	170.0 (8.5)	170.0 (14.0)	169.0 (10.0)	0.7	1.0
Weight (kg)	70.0 (16.0)	72.0 (18.0)	74.0 (24.0)	75.0 (20.0)	0.002	0.02
BMI (kg/m^2^)	24.6 (5.1)	25.7 (5.8)	25.2 (4.9)	27.4 (5.8)	0.003	0.009
**Medical history**						
Diabetes (n)	4 (2.4%)	4 (2.6%)	3 (6.1%)	1 (3.6%)	0.5	0.6
Hypertension (n)	74 (43.8%)	85 (55.6%)	34 (69.4%)	23 (82.1%)	< 0.001	< 0.001
Chronic obstructive pulmonary disease (n)	4 (2.4%)	6 (3.9%)	1 (2.0%)	0 (0.0%)	0.7	0.6
Previous myocardial infarction (n)	1 (0.6%)	0 (0.0%)	0 (0.0%)	0 (0.0%)	0.2	0.7
Peripheral vascular disease (n)	13 (7.7%)	11 (7.2%)	5 (10.2%)	1 (3.6%)	0.9	0.8
Smoking (n)	57 (33.7%)	59 (38.6%)	19 (38.8%)	12 (42.9%)	0.3	0.7
**Preoperative laboratory tests**						
Platelet (109/L)	197.0 (71.0)	186.5 (71.8)	199.0 (65.5)	166.5 (68.3)	0.006	0.01
Lymphocyte (109/L)	1.63 (0.74)	1.58 (0.90)	1.18 (0.72)	1.48 (0.90)	0.004	0.001
Monocyte (109/L)	0.44 (0.30)	0.51 (0.40)	0.79 (0.78)	0.91 (0.70)	< 0.001	< 0.001
Neutrophil (109/L)	4.22 (2.95)	4.86 (4.30)	7.51 (5.64)	10.1 (5.3)	< 0.001	< 0.001
Hyperlipidemia (n)	55 (34.4%)	44 (31.0%)	7 (14.9%)	10 (43.5%)	0.2	0.04
Retinol binding protein (mg/L)	35.7 (16.2)	37.8 (18.7)	26.4 (24.7)	37.3 (22.5)	0.7	0.007
Urinary protein						
+/−	17 (11.6%)	25 (18.8%)	10 (23.8%)	4 (20.0%)	0.05	0.2
+	14 (9.5%)	20 (15.0%)	11 (26.2%)	8 (40.0%)	0.01	0.005
++	1 (0.7%)	2 (1.5%)	2 (4.8%)	1 (5.0%)	0.2	0.3
INR	1.04 (0.12)	1.04 (0.1)	1.09 (0.15)	1.10 (0.2)	0.05	0.02
**Preoperative renal function**						
Preoperative SCr (μmol/L)	70.4 (21.79)	73.6 (23.1)	66.0 (26.3)	76.0 (45.7)	0.3	0.07
> 106 (n)	11 (6.5%)	13 (8.5%)	4 (8.2%)	8 (28.6%)	0.1	0.002
eGFR(mL/min/1.73 m2)	99.8 (22.0)	97.2 (21.2)	103.4 (18.2)	94.4 (39.5)	0.2	0.08
**Preoperative cardiovascular status**						
Marfan syndrome (n)	14 (8.3%)	3 (2.0%)	1 (2.0%)	1 (3.6%)	0.005	0.04
Aortic dissection (n)	56 (33.1%)	71 (46.4%)	36 (73.5%)	24 (85.7%)	< 0.001	< 0.001
Stanford type-A aortic dissection (n)	53 (31.4%)	71 (46.4%)	34 (69.4%)	24 (85.7%)	< 0.001	< 0.001
**Intraoperative blood product use**						
Erythrocytes (u)	2.0 (2.0)	4.0 (3.9)	4.0 (6.0)	4.0 (4.5)	0.001	< 0.001
Fresh frozen plasma (ml)	400.0 (200.0)	400.0 (150.0)	400.0 (375.0)	600.0 (500.0)	0.06	0.002
Platelets (u)	1.0 (0.0)	1.0 (1.0)	1.0 (1.0)	2.0 (1.0)	0.002	< 0.001
**Surgical details**						
Involving the aortic arch (n)	79 (46.7%)	97 (63.4%)	38 (77.6%)	26 (92.9%)	< 0.001	< 0.001
Involving the descending aorta (n)	43 (25.4%)	60 (39.2%)	33 (67.3%)	24 (85.7%)	< 0.001	< 0.001
CPB duration(min)	133.0 (75.5)	165.0 (79.0)	201.0 (67.5)	220.0 (52.0)	< 0.001	< 0.001
DHCA or MHCA (n)	58 (34.3%)	76 (49.7%)	34 (69.4%)	25 (89.3%)	< 0.001	< 0.001
Aortic cross-clamp time (min)	86.0 (27.0)	90.0 (31.0)	96.0 (32.0)	113.0 (32.0)	< 0.001	< 0.001
Operation time (h)	6.0 (2.0)	7.0 (2.0)	8.5 (2.0)	9.0 (2.5)	< 0.001	< 0.001
Combined surgery						
Valvular surgery (n)	94 (55.6%)	67 (43.8%)	10 (20.4%)	3 (10.7%)	< 0.001	< 0.001
**Outcomes**						
Renal replacement therapy (n)	0 (0.0%)	0 (0.0%)	0 (0.0%)	12 (42.9%)	0.003	< 0.001
Length of ICU stay (day)	3.0 (2.0)	4.0 (2.5)	5.0 (4.0)	10.5 (11.0)	< 0.001	< 0.001
> 7 d (n)	19 (11.2%)	20 (13.1%)	17 (34.7%)	23 (82.1%)	< 0.001	< 0.001
Intubation time (h)	15.0 (9.0)	17.0 (27.5)	38.0 (72.5)	136.0 (110.3)	< 0.001	< 0.001
> 5 d (n)	4 (2.4%)	5 (3.3%)	6 (12.2%)	17 (60.7%)	< 0.001	< 0.001
Stroke (n)	0 (0.0%)	4 (2.6%)	0 (0.0%)	2 (7.1%)	0.04	0.01
Redo surgery (n)	4 (2.4%)	6 (3.9%)	6 (12.2%)	6 (21.4%)	0.02	< 0.001
In-hospital mortality (n)	2 (1.2%)	1 (0.7%)	1 (2.1%)	8 (28.6%)	0.07	< 0.001

Abbreviations: *BMI* body mass index, *INR* International Normalized Ratio, *SCr* Serum Creatinine, *eGFR* Estimated Glomerular Filtration Rate, *CPB* Cardiopulmonary Bypass, *DHCA* Deep Hypothermia and Circulatory Arrest, *MHCA* Median Hypothermia and Circulatory Arrest

NOTE. The categorical variables in the table are presented by the number of cases (with percentage) and the continuous variables are expressed by the median (with interquartile range) or mean (with standard deviation).

^*^P1 Value: Compare the patients without AKI and all patients with AKI. *P*_1_ values* were the results of unpaired t-test or Mann-Whitney U test for continuous variables, and χ2 test or Fisher’s exact test for categorical variables.

^#^*P*2 Value: Compare patients within four groups (without AKI and patients with stage 1, 2 and 3 AKI). *P*_2_ values# were the results of one-way analysis of variance or KruskalWallis test for continuous variables, and χ2 test or Fisher’s exact test for categorical variables

#### Postoperative AKI in the patients with AAS

In the subgroup of patients with AAS, the incidence of postoperative AKI was evidently higher than that of the overall cohort (*p* < 0.01). 131 in 187 (70.1%) developed the postoperative AKI, among whom 71 patients (38.0%) met the criteria of stage 1, 36 patients (19.3%) satisfied the criteria for stage 2 AKI, and 24 patients (12.8%) met reached the stage 3 (Table 3).

#### Postoperative AKI in the patients without AAS

Compared with the AAS subgroup, the incidence of postoperative AKI was markedly reduced in the non-AAS subgroup (*p* < 0.001). Of 212 patients in the non-AAS subgroup, 99 (46.7%) patients developed the postoperative AKI. 82 patients (38.7%) met the criteria of stage 1, 13 patients (6.1%) reached the criteria for stage 2, 4 patients (1.9%) were in stage 3 (Table 5).

### Risk factors of postoperative AKI after open thoracic aortic surgery

#### Risk factors of postoperative AKI for the overall cohort

Demographic and perioperative data of patients grouped by the severity of the KDIGO classification were present in Table 1. There were significant statistical differences in a series of variables including age, weight, BMI, history of hypertension, preoperative platelet, lymphocyte, monocyte, neutrophil and several other laboratory variables, Marfan syndrome, aortic dissection (as well as the Stanford type-A aortic dissection), intraoperative infusion of erythrocytes, fresh frozen plasma and platelets, combined valvular surgery and all the surgical data (Table 1).

The incidences of CRRT, ICU stay > 7 days, intubation time > 5 days, stroke, redo surgery and in-hospital mortality were all significantly higher among the patients with AKI than those without AKI. The time of ICU stay and intubation also markedly increased in the patients with AKI compared those without AKI (Table 1).

In the univariate analysis of risk factors for the overall cohort, body weight, history of hypertension, Marfan syndrome, preoperative platelet, lymphocyte, monocyte and neutrophil, neutrophil to lymphocyte ratio (NLR), lymphocyte to monocyte ratio (LMR), preoperative urinary protein positive (+,++), Marfan syndrome, aortic dissection (Stanford type-A aortic dissection), intraoperative fresh frozen plasma use, intraoperative involvement of aortic arch and descending aorta, CPB time, aortic cross-clamp time, operation time, DHCA or MHCA and concurrent valve surgery were candidate variables (Table 2).

**Table 2:** Univariate Analysis of Risk Factors for the Overall Cohort

Variable	Odds Ratio	95% Confidence Interval	*P* Value
Age (y)	1.01	0.99–1.02	0.6
Sex, male	1.48	0.95–2.28	0.08
Weight	1.02	1.01–1.04	0.006
Hypertension	2.07	1.38–3.10	< 0.001
Preoperative platelet	1.00	0.99–1.00	0.006
Preoperative lymphocyte	0.64	0.46–0.88	0.007
Preoperative monocyte	3.76	2.02–7.00	< 0.001
Preoperative neutrophil	1.16	1.08–1.23	< 0.001
NLR	1.17	1.10–1.25	< 0.001
LMR	0.88	0.81–0.96	0.003
Preoperative urinary protein + or ++	2.43	1.30–4.53	0.005
Marfan syndrome	0.25	0.09–0.70	0.008
Aortic dissection	2.67	1.77–4.04	< 0.001
Standford type-A aortic dissection	2.80	1.84–4.24	< 0.001
Intraoperative fresh frozen plasma use	1.00	1.00–1.00	0.01
Involving the aortic arch	2.66	1.76–4.02	< 0.001
Involving the descending aorta	3.03	1.97–4.67	< 0.001
CPB duration	1.01	1.01–1.02	< 0.001
DHCA or MHCA	2.72	1.80–4.11	< 0.001
Aortic cross-clamp time	1.02	1.01–1.02	< 0.001
Operation time	1.63	1.41–1.89	< 0.001
Combined valvular surgery	0.43	0.28–0.64	< 0.001

Abbreviations: *NLR* Neutrophil to Lymphocyte Ratio, *LMR* Lymphocyte to Monocyte Ratio, *SCr* Serum Creatinine, *CPB* Cardiopulmonary Bypass, *DHCA* Deep Hypothermia and Circulatory Arrest, *MHCA* Median Hypothermia and Circulatory Arrest

#### Risk factors of postoperative AKI for the patients with AAS

For the subgroup of patients with AAS, there existed significant statistical differences in those variables including male gender, weight, history of hypertension, preoperative lymphocyte, monocyte and neutrophil and several other laboratory variables, CPB time, aortic cross-clamp time, operation time and combined CABG (Table 3).

**Table 3:** Characteristics of the patients with acute aortic syndrome

	No AKI	AKI Stage			*P*1 Value^*^	*P*2 Value^#^
		1	2	3		
Patient population (n)	56	71	36	24		
**Demographic data**						
Age (y)	48.0 (16.5)	48.0 (15.0)	48.0 (13.5)	41.0 (8.3)	0.2	0.07
Sex, male (n)	31 (55.4%)	52 (73.2%)	24 (66.7%)	18 (75.0%)	0.03	0.1
Height (cm)	170.0 (12.8)	171.0 (7.0)	172.0 (10.5)	170.0 (13.0)	0.4	0.5
Weight (kg)	71.9 ± 10.2	78.0 ± 12.6	77.1 ± 14.4	77.8 ± 13.4	0.003	0.07
BMI (kg/m^2^)	25.5 ± 2.8	25.7 ± 5.6	26.4 ± 3.9	28.2 ± 3.7	0.3	0.2
**Medical history**						
Diabetes (n)	2 (3.6%)	1 (1.4%)	2 (5.6%)	1 (4.2%)	0.9	0.7
Hypertension (n)	38 (67.9%)	46 (64.8%)	30 (83.3%)	23 (95.8%)	0.3	0.009
Chronic obstructive pulmonary disease (n)	1 (1.8%)	1 (1.4%)	0 (0.0%)	0 (0.0%)	0.5	0.8
Peripheral vascular disease (n)	4 (7.1%)	5 (7.0%)	4 (11.1%)	1 (4.2%)	0.9	0.8
Smoking (n)	17 (30.4%)	21 (29.6%)	13 (36.1%)	9 (37.5%)	0.7	0.8
**Preoperative laboratory tests**						
Platelet (109/L)	195.5 (106.0)	191.0 (82.0)	191.0 (83.3)	177.0 (65.5)	0.1	0.2
Lymphocyte (109/L)	1.39 (0.75)	1.24 (0.81)	1.13 (0.59)	1.41 (0.99)	0.01	0.03
Monocyte (109/L)	0.70 (0.56)	0.67 (0.60)	0.89 (0.63)	0.92 (0.82)	0.03	0.03
Neutrophil (109/L)	7.39 ± 3.60	7.76 ± 3.41	8.97 ± 3.33	10.52 ± 3.76	0.04	0.001
Hyperlipidemia (n)	14 (25.0%)	16 (22.5%)	3 (8.3%)	9 (37.5%)	0.6	0.02
Retinol binding protein (mg/L)	27.6 (24.1)	27.9 (17.1)	21.2 (16.4)	37.3 (25.2)	0.7	0.02
**Preoperative renal function**						
Dissection involving renal artery (n)	4 (7.1%)	6 (8.5%)	4 (11.1%)	5 (20.8%)	0.4	0.3
Preoperative SCr (μmol/L)	65.5 (31.0)	75.0 (24.4)	68.5 (36.2)	81.1 (47.9)	0.2	0.2
> 106 (n)	6 (10.7%)	10 (14.1%)	4 (11.1%)	7 (29.2%)	0.3	0.2
**Surgical details**						
Involving the aortic arch (n)	50 (89.3%)	66 (93.0%)	35 (97.2%)	24 (100.0%)	0.1	0.2
Involving the descending aorta (n)	43 (76.8%)	59 (83.1%)	33 (91.7%)	23 (95.8%)	0.06	0.1
CPB duration (min)	188.0 (38.8)	201.0 (44.0)	222.0 (51.8)	235.0 (60.0)	< 0.001	< 0.001
DHCA or MHCA (n)	48 (85.7%)	65 (91.5%)	34 (94.4%)	23 (95.8%)	0.1	0.4
Aortic cross-clamp time (min)	94.5 (21.8)	99.0 (31.0)	96.0 (32.5)	110.0 (29.0)	0.02	0.02
Operation time (h)	7.0 (1.5)	8.0 (1.5)	8.5 (1.9)	9.0 (2.5)	< 0.001	< 0.001
Combined surgery						
CABG (n)	1 (1.8%)	2 (2.8%)	0 (0.0%)	3 (12.5%)	0.5	0.04
Valvular surgery (n)	8 (14.3%)	7 (9.9%)	1 (2.8%)	0 (0.0%)	0.07	0.1
**Outcomes**						
Renal replacement therapy (n)	0 (0.0%)	0 (0.0%)	0 (0.0%)	11 (45.8%)	0.03	< 0.001
Length of ICU stay (day)	5.0 (3.8)	5.0 (2.0)	5.0 (4.0)	12.0 (11.5)	0.02	< 0.001
> 7 d (n)	17 (30.4%)	16 (22.5%)	13 (36.1%)	22 (91.7%)	0.3	< 0.001
Intubation time (h)	19.0 (20.0)	24.0 (32.0)	40.5 (75.8)	147.5 (81.0)	< 0.001	< 0.001
> 5 d (n)	4 (7.1%)	4 (5.6%)	5 (13.9%)	17 (70.8%)	0.03	< 0.001
Redo surgery (n)	2 (3.6%)	2 (2.8%)	5 (13.9%)	6 (25.0%)	0.1	0.002
In-hospital mortality (n)	1 (1.8%)	1 (1.4%)	0 (0.0%)	7 (29.2%)	0.2	< 0.001

Abbreviations: *BMI* body mass index, *SCr* Serum Creatinine, *CPB* Cardiopulmonary Bypass, *DHCA* Deep Hypothermia and Circulatory Arrest, *MHCA* Median Hypothermia and Circulatory Arrest, *CABG* Coronary Artery Bypass Graft

NOTE. The categorical variables in the table are presented by the number of cases (with percentage) and the continuous variables are expressed by the median (with interquartile range) or mean (with standard deviation)

^*^P1 Value: Compare the patients without AKI and all patients with AKI. *P*_1_ values* were the results of unpaired t-test or Mann-Whitney U test for continuous variables, and χ2 test or Fisher’s exact test for categorical variables

^#^*P*2 Value: Compare patients within four groups (without AKI and patients with stage 1, 2 and 3 AKI). *P*_2_ values# were the results of one-way analysis of variance or KruskalWallis test for continuous variables, and χ2 test or Fisher’s exact test for categorical variables

Similarly as above, the proportions of CRRT, ICU stay > 7 days, intubation time > 5 days, stroke, redo surgery and in-hospital mortality were all higher in the AKI group. Consistently, the durations of ICU stay and intubation were evidently increased in the patients with AKI in comparison with those without AKI (Table 3).

When it comes to the univariate analysis of risk factors for the AAS cohort, male gender, weight, preoperative lymphocyte, monocyte, neutrophil, NLR, LMR, CPB time, aortic cross-clamp time and operation time were included in the list of plausible variables (Table 4).

**Table 4:** Univariate Analysis of Risk Factors for the patients with acute aortic syndrome

Variable	Odds Ratio	95% Confidence Interval	*P* Value
Age (y)	0.98	0.95–1.01	0.2
Sex, male	2.05	1.07–3.92	0.03
Weight	1.04	1.01–1.07	0.009
Preoperative platelet	1.00	0.99–1.00	0.06
Preoperative lymphocyte	0.61	0.37–1.01	0.05
Preoperative monocyte	2.42	1.10–5.36	0.03
Preoperative neutrophil	1.10	1.01–1.21	0.04
NLR	1.16	1.06–1.28	0.002
LMR	0.77	0.64–0.93	0.007
Preoperative NYHA class III or IV	0.39	0.14–1.03	0.06
Involving the descending aorta	2.17	0.97–4.89	0.06
CPB duration	1.02	1.01–1.03	< 0.001
DHCA or MHCA	2.26	0.82–6.20	0.1
Aortic cross-clamp time	1.02	1.00–1.04	0.01
Operation time	1.63	1.26–2.10	< 0.001
Combined valvular surgery	0.39	0.14–1.1	0.08

Abbreviations: *NLR* Neutrophil to Lymphocyte Ratio, *LMR* Lymphocyte to Monocyte Ratio, *CPB* Cardiopulmonary Bypass, *DHCA* Deep Hypothermia and Circulatory Arrest, *MHCA* Median Hypothermia and Circulatory Arrest

#### Risk factors of postoperative AKI for the patients without AAS

For the non-AAS subgroup, those variables reached statistical significance that include age, history of hypertension, preoperative platelet and eGFR, previous cardiovascular surgery, Marfan syndrome, intraoperative perfusion of fresh frozen plasma and platelets, operation involving the descending aorta, CPB time, aortic cross-clamp time, DHCA or MHCA and operation time (Table 5).

**Table 5:** Characteristics of the patients without acute aortic syndrome

	No AKI	AKI Stage			*P*1 Value^*^	*P*2 Value^#^
		1	2	3		
**Patient population (n)**	113	82	13	4		
**Demographic data**						
Age (y)	53.0 (17.5)	60.0 (11.0)	52.0 (14.0)	55.0 (19.5)	0.002	0.01
Sex, male (n)	81 (71.7%)	64 (78.0%)	10 (76.9%)	3 (75.0%)	0.3	0.8
Height (cm)	170.0 (14.0)	168.0 (10.5)	165.0 (15.0)	166.5 (4.5)	0.1	0.3
Weight (kg)	67.0 (16.0)	69.1 (15.6)	66.0 (15.0)	62.0 (17.0)	0.6	0.2
BMI (kg/m^2^)	24.2 (5.3)	25.0 (5.7)	22.9 (5.0)	22.8 (5.6)	0.09	0.09
**Medical history**						
Diabetes (n)	2 (1.8%)	3 (3.7%)	1 (7.7%)	0 (0.0%)	0.3	0.6
Hypertension (n)	36 (31.9%)	39 (47.6%)	4 (30.8%)	0 (0.0%)	0.08	0.05
Chronic obstructive pulmonary disease (n)	3 (2.7%)	5 (6.1%)	1 (7.7%)	0 (0.0%)	0.2	0.6
Previous myocardial infarction (n)	1 (0.9%)	0 (0.0%)	0 (0.0%)	0 (0.0%)	0.3	0.8
Peripheral vascular disease (n)	9 (8.0%)	6 (7.3%)	1 (7.7%)	0 (0.0%)	0.8	0.9
Smoking (n)	40 (35.4%)	38 (46.3%)	6 (46.2%)	3 (75.0%)	0.08	0.2
**Preoperative laboratory tests**						
Platelet (109/L)	197.0 (57.5)	183.0 (58.0)	203.0 (54.5)	137.5 (109.3)	0.03	0.09
**Preoperative renal function**						
eGFR(mL/min/1.73 m2)	99.8 (16.1)	95.9 (18.1)	101.7 (16.4)	97.2 (37.6)	0.04	0.02
**Preoperative cardiovascular status**						
Previous cardiovascular surgery (n)	2 (1.8%)	1 (1.2%)	0 (0.0%)	1 (25.0%)	0.9	0.008
LVEF (%)	58.0 (8.0)	60.0 (4.5)	52.5 (17.8)	59.0 (11.8)	0.2	0.08
Marfan syndrome (n)	11 (9.7%)	2 (2.4%)	1 (7.7%)	0 (0.0%)	0.05	0.2
**Intraoperative blood product use**						
Erythrocytes (u)	2.0 (3.0)	2.0 (4.0)	3.0 (3.0)	4.0 (6.0)	0.2	0.07
Fresh frozen plasma (ml)	400.0 (0.0)	400.0 (0.0)	400.0 (300.0)	650.0 (450.0)	0.2	0.02
Platelets (u)	1.0 (1.0)	1.0 (1.0)	1.0 (1.0)	2.0 (0.8)	0.5	0.004
**Surgical details**						
Involving the aortic arch (n)	29 (25.7%)	31 (37.8%)	3 (23.1%)	2 (50.0%)	0.09	0.2
Involving the descending aorta (n)	0 (0.0%)	1 (1.2%)	0 (0.0%)	1 (25.0%)	0.1	< 0.001
CPB duration(min)	117.5 (43.0)	131.0 (50.5)	137.0 (52.5)	187.5 (34.8)	0.005	0.003
DHCA or MHCA (n)	10 (8.8%)	11 (13.4%)	0 (0.0%)	2 (50.0%)	0.3	0.03
Aortic cross-clamp time (min)	81.0 (27.8)	83.0 (31.3)	94.0 (32.5)	122.0 (42.8)	0.08	0.04
Operation time (h)	5.0 (1.0)	6.0 (1.5)	7.0 (2.3)	8.5 (2.5)	< 0.001	< 0.001
Combined surgery						
CABG (n)	8 (7.1%)	11 (13.4%)	2 (15.4%)	1 (25.0%)	0.09	0.3
**Outcomes**						
Renal replacement therapy (n)	0 (0.0%)	0 (0.0%)	0 (0.0%)	1 (25.0%)	0.3	< 0.001
ICU length of stay (day)	3.0 (1.0)	3.0 (2.0)	5.0 (5.0)	4.0 (7.5)	< 0.001	< 0.001
> 7 d (n)	2 (1.8%)	4 (4.9%)	4 (30.8%)	1 (25.0%)	0.02	< 0.001
Intubation time (h)	13.0 (8.0)	14.0 (9.0)	20.0 (28.5)	28.5 (28.5)	0.03	0.001
> 5 d (n)	0 (0.0%)	1 (1.2%)	1 (7.7%)	0 (0.0%)	0.1	0.06
In-hospital mortality (n)	1 (0.9%)	0 (0.0%)	1 (7.7%)	1 (25.0%)	0.5	< 0.001

Abbreviations: *BMI* body mass index, *INR* International Normalized Ratio, *eGFR* Estimated Glomerular Filtration Rate, *LVEF* Left Ventricular Ejection Fraction, *CPB* Cardiopulmonary Bypass, *DHCA* Deep Hypothermia and Circulatory Arrest, *MHCA* Median Hypothermia and Circulatory Arrest, *CABG* Coronary Artery Bypass Graft

NOTE. The categorical variables in the table are presented by the number of cases (with percentage) and the continuous variables are expressed by the median (with interquartile range) or mean (with standard deviation)

^*^P1 Value: Compare the patients without AKI and all patients with AKI. *P*_1_ values* were the results of unpaired t-test or Mann-Whitney U test for continuous variables, and χ2 test or Fisher’s exact test for categorical variables

^#^*P*2 Value: Compare patients within four groups (without AKI and patients with stage 1, 2 and 3 AKI). *P*_2_ values# were the results of one-way analysis of variance or KruskalWallis test for continuous variables, and χ2 test or Fisher’s exact test for categorical variables

For the patients in the non-AAS subgroup, the rates of CRRT, ICU stay > 7 days, intubation time > 5 days and in-hospital mortality were evidently enhanced when compared to those without AKI. And the ICU length of stay also elevated in the patients with AKI (Table 5).

As for the univariate analysis of risk factors for the non-AAS cohort, several potential risk factors have been identified that consist of age, preoperative platelet and platelet to lymphocyte ratio (PLR), CPB time and operation time (Table 6).

**Table 6:** Univariate Analysis of Risk Factors for the patients without acute aortic syndrome

Variable	Odds Ratio	95% Confidence Interval	*P* Value
Age (y)	1.05	1.02–1.08	< 0.001
Sex, male	1.38	0.74–2.59	0.3
Hypertension	1.64	0.94–2.88	0.08
Smoking	1.65	0.95–2.86	0.08
Preoperative platelet	0.99	0.99–1.00	0.02
PLR	0.99	0.99–1.00	0.05
Preoperative eGFR	0.98	0.97–1.00	0.06
Marfan syndrome	0.29	0.08–1.07	0.06
Involving the aortic arch	1.66	0.92–2.98	0.09
CPB duration	1.01	1.00–1.02	0.01
Operation time	1.56	1.22–1.99	< 0.001

Abbreviations: *PLR* Platelet to Lymphocyte Ratio, *eGFR* Estimated Glomerular Filtration Rate, *CPB* Cardiopulmonary Bypass

### The establishment of predictive model for postoperative AKI

#### Predictive model for postoperative AKI in the overall cohort

**Fig. 2:**
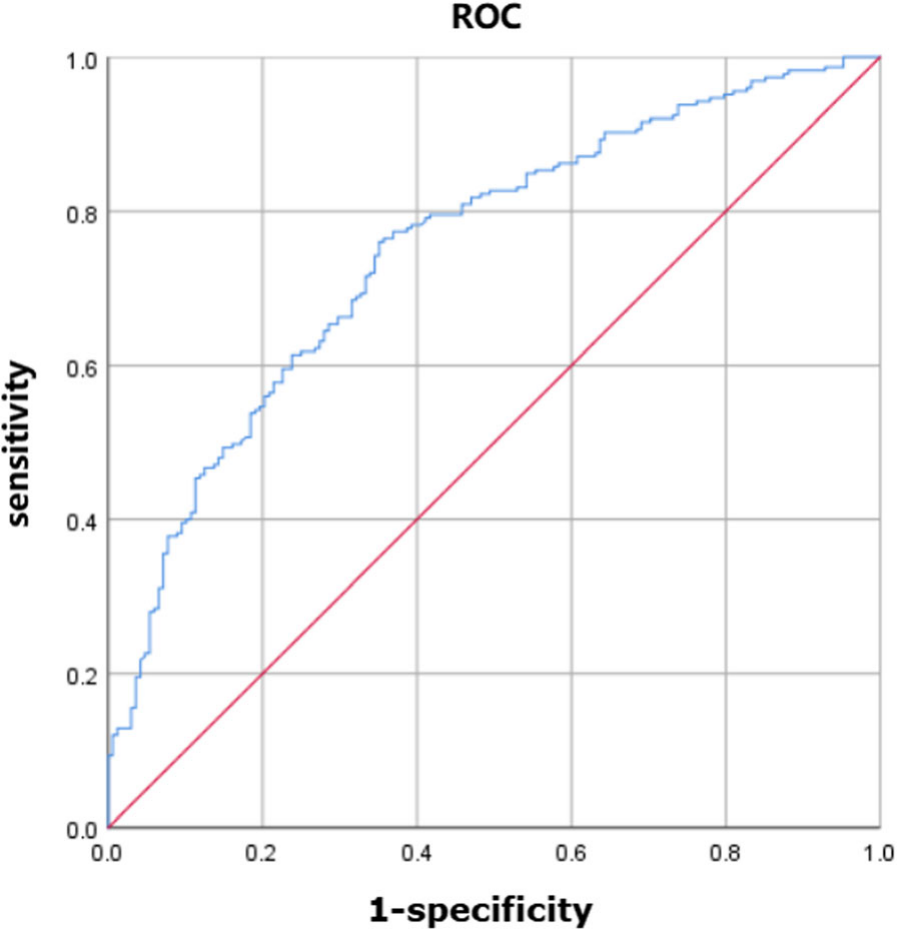
ROC curves of predictive models of acute kidney injury in the overall cohort. Figure 2 shows the ROC curves of predictive models of acute kidney injury in the overall cohort

Those independent predictors of postoperative AKI after OTAS included history of hypertension (OR 1.011, 95% CI: [1.001–1.022], *p* = 0.04), preoperative platelet (OR 0.995, 95% CI: [0.991–0.999], *p* = 0.006) and operation time (OR 1.572, 95% CI: [1.355–1.823], *p* < 0.001). The equation obtained by binary Logistic regression analysis was as follows: Y = 0.011 × history of hypertension-0.005 × preoperative platelet+ 0.452 × operation time-4.814. The predictive model was evaluated by the ROC curve. The area under a curve (AUC) of the ROC curve was 0.750 (*p* < 0.001, 95%CI: 0.702–0.798) (Fig. 2). When the maximum value of the Youden index was 0.409, at this time Y = 0.031, the sensitivity of the model was 76.0% and the specificity was 64.9%.

#### Predictive model for postoperative AKI in the patients with AAS

**Fig. 3:**
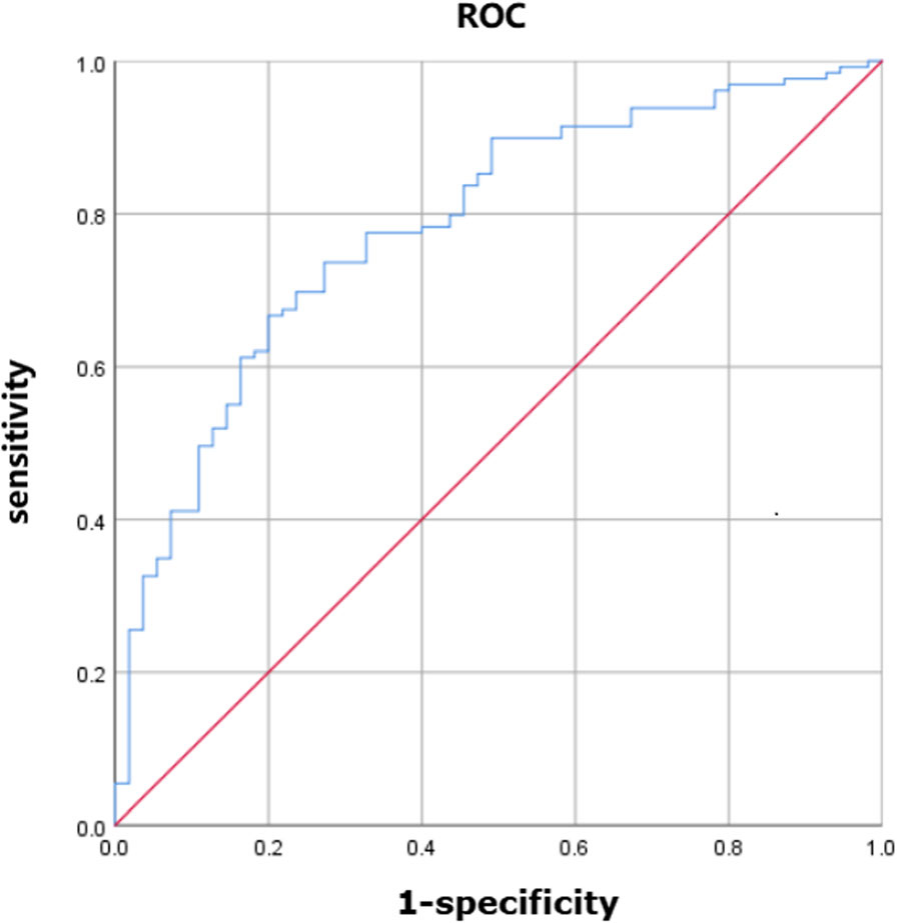
ROC curves of predictive models of acute kidney injury in the AAS subgroup. Figure 3 shows the ROC curves of predictive models of acute kidney injury in the AAS subgroup

For the patients with AAS, CPB time (OR 1.020, 95% CI: [1.009–1.031], *p <* 0.001) and preoperative LMR (OR 0.823, 95% CI: [0.701–0.966], *p* = 0.02) independently predicted the incidence of postoperative AKI. The equation obtained by binary Logistic regression analysis was: Y = 0.020 × CPB time-0.195 × LMR-5.601. The AUC of ROC curve was 0.784 (*p <* 0.001, 95% CI: 0.714–0.855) (Fig. 3). When the maximum value of the Youden index reached 0.467, at this time Y = 1.056, the sensitivity of the model was 66.7% and the specificity was 80.0%.

#### Predictive model for postoperative AKI in the patients without acute aortic syndrome

**Fig. 4:**
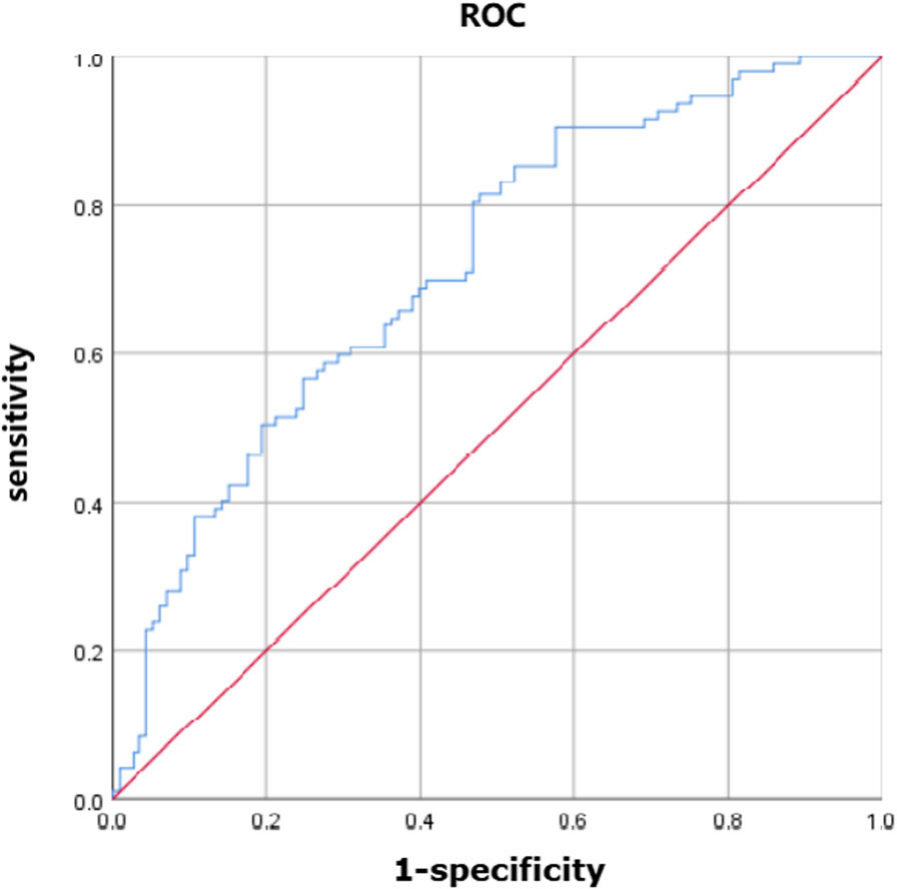
ROC curves of predictive models of acute kidney injury in the non-AAS subgroup. Figure 4 shows the ROC curves of predictive models of acute kidney injury in the non-AAS subgroup

For the patients without AAS, age (OR 1.045, 95% CI: [1.015–1.076], *p* = 0.003), preoperative platelet (OR 0.993, 95% CI: [0.988–0.998], *p* = 0.04) and operation time (OR 1.496, 95% CI: [1.166–1.918], *p* = 0.002) independently predicted the incidence of postoperative AKI. The equation obtained by binary Logistic regression analysis was: Y = 0.044 × age-0.006 × preoperative platelet+ 0.403 × operation time-3.751. The AUC of ROC curve (see Fig. 4) was 0.719 (*P* < 0.001, 95% CI: 0.651–0.787). When the maximum value of the Youden index reached 0.467, at this time Y = 1.0564, the sensitivity of the model was 81.4% and the specificity was 52.2%.

## Discussion

Prospective or retrospective studies have previously found that the incidence of AKI after thoracic aortic surgery is approximately 18–67%, depending on the definition of AKI [1–7]. Some of these prior papers recruited a highly heterogeneous group of patients, regardless of disease types and surgical options. And the other papers were designed to study on a specific group of patients, such as the patients undergoing elective or emergent OTAS, overweight patients who underwent total aorta replacement (TAR) combined with frozen elephant trunk (FET) implantation, patients receiving surgical treatment for type A aortic dissection and so on. We believe that the deviation of AKI incidence and predictive factors from various studies on OTAS was to a large extent attributable to the heterogeneity of recruited subjects including ethnicity, disease types and surgical procedures and etc.

According to our search result, the incidence of postoperative AKI following OTAS has been rarely studied in the patients with AAS and our work was among the first to focus on this special subgroup. The definition of AAS comprises of a series of acute and catastrophic aortic lesions including acute aortic dissection, intramural hematoma, penetrating aortic ulcer, and rupture of aorta due to aortic aneurysm or trauma [14]. For most heart centers across the globe including ours, emergency aortic surgery is indicated for the patients with AAS. Even for the patients at advanced age and with co-morbidities, urgent surgery is highly recommended, and for the most circumstances is not surgically contraindicated. Of note, the patients with AAS are theoretically under a higher risk of postoperative AKI than the overall cohort and non-AAS group. First, the patients with AAS are more likely to develop into a serious situation of single- or multi-organ dysfunction including cardiac, hepatic and respiratory failure, which is tightly associated with renal dysfunction and even failure. Second, acute aortic lesion could lead to the sudden disruption of normal blood supply to renal, which inevitably results in the occurrence of AKI. Third, the systemic inflammation response syndrome (SIRS) and ischemia-reperfusion injury could further deteriorate the status of renal function and worsen the AKI. Other preoperative risk factors among AAS patients compared to non-AAS patients might include hypertension, obesity, preoperative renal injury and repeated contrast agents used in CT or MRI scan. Fourth, a majority of patients with AAS will undergo the aortic arch repair and reconstruction, which signifies the prolonged operation time, CPB time and DHCA or MHCA. Especially in China, the patients with AAS have a younger average age at disease onset than the patients in the western countries [15]. And the Chinese patients prefer to undergo a more radical surgical operation (such as Sun’s procedure) for only once because of limited financial support and medical insurance. Therefore, the patients with AAS should be separately analyzed for the incidence and risk factors of postoperative AKI following OTAS. Our results revealed that compared to the non-AAS subgroup, an extra of 15% more patients with AAS will experience the AKI following OTAS. And the prolonged CPB time and reduced preoperative LMR were independently associated with the occurrence of AKI in the AAS subgroup. As expected, the predictive models for AKI were different between the AAS and non-AAS subgroups. Our results were consistent with several previous studies that CPB time instead of DHCA time were statistically linked with postoperative AKI [7, 15]. It is hypothesized by Sun and this team that DHCA could significantly extend the CPB time, which is likely to be counterbalanced by the protective effects of DHCA on renal function. Additionally, the preoperative LMR might also function as a novel bio-marker for predicting postoperative AKI following OTAS, and such report has not been published so far and it awaits further investigation.

Our team also analyzed the data from the non-AAS subgroup in whom elective OTAS were performed. Englberger and his colleagues carried out a retrospective study with a larger sample size on elective OTAS [2]. However, our work was the first to investigate the AKI following elective OTAS in Chinese ethnicity though the sample size was relatively small. Besides, the results of diagnostic test suggested that our predictive model for AKI after OTAS has a good predictive ability, with its sensitivity and specificity to be further improved in the future.

## Conclusions

The patients with AAS have a higher risk of developing postoperative AKI after OTAS compared with those without AAS. The predictive factors for postoperative AKI following OTAS vary between AAS and non-AAS subgroups. Operation time, CPB time and preoperative platelet are modifiable predictors of AKI.

## Supplementary information

**Additional file 1.**

**Additional file 2.** Supplementary Table 1 Surgical options of the overall cohort

**Additional file 3.** Supplementary Table 2 Surgical options of AAS patient

**Additional file 4.** Supplementary Table 3 Surgical options of non-AAS patients

## Data Availability

The datasets used and/or analyzed during the current study are available from the corresponding author on reasonable request.

## Abbreviations

AKI: Acute kidney injury
OTAS: Open thoracic aortic surgery
AAS: Acute aortic syndrome
CPB: Cardiopulmonary bypass
RRT: Renal replacement therapy
GCP: Good Clinical Practice
KDIGO: Kidney Disease: Improving Global Outcomes guidelines
Scr: Serum creatinine
SD: Standard deviation
ROC: Receiver operating curve
NLR: Neutrophil to lymphocyte ratio
LMR: Lymphocyte to monocyte ratio
PLR: Platelet to lymphocyte ratio
TAR: Total aorta replacement
FET: Frozen elephant trunk
SIRS: Systemic inflammation response syndrome
