# Supplementary material for: Risk factors analysis of acute kidney injury following open thoracic aortic surgery in the patients with or without acute aortic syndrome: a retrospective study
Source: J Cardiothorac Surg. 2020 Aug 7;15:213. doi: 10.1186/s13019-020-01257-1 (PMC7412815; doi:10.1186/s13019-020-01257-1)
Supplement: Supplementary file 1 — Additional file 1. [file 13019_2020_1257_MOESM1_ESM.docx]

Supplementary Materials

**Surgical details**

A median sternotomy was performed under general anesthesia. Anesthesia was maintained either by total intravenous anesthetics (propofol, vecuronium bromide and sufentanil) or inhalation agent (sevoflurane). Tranexamic acid was used for coagulation support. CPB was instituted routinely at 2.2 to 2.5 L/min/m^2^. When the lesion involves the aortic arch, arterial cannulation was positioned in the right axillary and/or femoral artery; venous cannulations were bicaval through right atrium. The cold blood cardioplegia for myocardial protection was perfused through the left and right coronary arteries. Repair and reconstruction of the aortic arch were performed under deep or moderate hypothermic and circulatory arrest (DHCA or MHCA), and selective antegrade cerebral perfusion via the right axillary artery. During the core cooling, accompanying cardiac procedures including aortic valve repair or replacement, sinus reconstruction, and root replacement were performed if necessary.

**The basic characteristics of overall cohort**

A total of 399 patients were enrolled in this retrospective observational cohort, amongst whom 70.9% were male with a mean age of 52.0(16.0) years. The mean arterial systolic pressure was 137.0(28.0) mmHg with the mean diastolic pressure being 76.0(20.0) mmHg, and the mean arterial pressure (MAP) being 95.0(19.7) mmHg. 63.2% of the patients were not smokers, and 54.1% were diagnosed with hypertension. Other comorbidities included the diabetes (3.0%), peripheral vascular disease (7.5%) and chronic pulmonary disease (2.8%). 1 patient (0.3%) had recent myocardial infarction and 9 patients (2.3%) had a prior history of cardiovascular surgery. 3 patients (0.8%) were admitted with gestational status. 19 patients (4.8%) had the Marfan syndrome and 168 cases (42.1%) were diagnosed with the ascending aorta dilatation or aneurysm. A total of 160 cases (40.1%) were diagnosed with DeBakay type I aortic dissection (AD), 22 cases (5.5%) with DeBakay type II AD, and 2 cases (0.5%) with DeBakay type III AD. 38 patients (9.5%) were diagnosed with aortic root aneurysm, 4 patients (1.0%) with thoracic aortic aneurysm, 3 patients (0.8%) with the aortic intermural hematoma (IMH), 1 patient (0.3%) with the aneurysm of brachiocephalic trunk, and 1 patient (0.3%) with the arteritis. Preoperative laboratory examinations showed that the serum creatinine (SCr) was 70.9(25.0)μmol/L, and the estimated glomerular filtration rate (eGFR) being 99.0(22.5)ml/min/1.73m^2^. 36 cases (9.0%) had a preoperative SCr>1.2mg/dl. The mean LVEF before operation was 60.0%(5.0%).

The surgical options of overall cohort were presented in the supplementary table 1. A sum of 240 cases (60.2%) underwent the aortic arch surgery, and 163 cases (40.9%) underwent the operations for descending aorta. During aortic surgery, 28 patients (7.0%) underwent CABG, 63 patients (15.8%) underwent valve replacement or shaping, and 3 patients (0.8%) underwent cesarean section. Mean aortic cross-clamp time was 91.0(32.0)min and mean CPB time was 165.0(86.0)min. A total of 193 cases (48.4%) experienced deep hypothermia and circulatory arrest (DHCA) or moderate hypothermia and circulatory arrest (MHCA).

12 patients (3.0%) received CRRT after surgery and 22 patients (5.5%) underwent a secondary surgery during hospitalization. 12 patients (3.0%) died during hospitalization.

**The basic characteristics of patients with acute aortic syndrome**

187 patients with acute aortic syndrome (AAS) were recruited in the AAS subgroup, amongst whom 66.8% were male, with a mean age of 47.0(14.0) years. The mean systolic blood pressure was 142.0(33.0) mmHg, with the mean diastolic blood pressure being 80.0(18.0) mmHg, and the MAP being 98.3(20.0)mmHg. 67.9% of patients were not smokers and 73.3% were diagnosed with hypertension. Other comorbidities included diabetes (3.2%), peripheral vascular disease (7.5%), and chronic pulmonary disease (1.1%). 5 patients (2.7%) had a prior history of cardiovascular surgery, and 3 patients (1.6%) were admitted with gestational status. 5 patients (2.7%) had Marfan syndrome. A total of 160 cases (85.6%) were diagnosed with DeBakey t ype I aortic dissection (AD), 22 cases (11.8%) with DeBakay type II AD, 2 cases (1.1%) with DeBakay type III AD, and 3 cases (1.6%) with IMH. Preoperative laboratory examinations demonstrated that the mean SCr of the AAS subgroup was 70.9(31.2) μmol/L and the mean the estimated eGFR was 99.7(26.4) ml/min/1.73m^2^. 27 cases (14.4%) had a preoperative SCr>1.2mg/dl. And the mean LVEF was 60.0%(2.0%).

The surgical options of patients with AAS were presented in the supplementary table 2. The majority (93.6%) of patients with AAS underwent aortic arch surgery and 158 patients (84.5%) underwent the operations for descending aorta. 17 cases (9.1%) underwent the ascending aorta and right aortic arch replacement, 6 cases (3.2%) underwent the Bentall procedure, 5 cases (2.7%) underwent the improved Cabrol surgery, and 1 case (0.5%) underwent the ascending aorta replacement. Concomitantly, 6 patients (3.2%) underwent CABG, 16 patients (8.6%) underwent valve replacement or shaping, and 3 patients (1.6%) underwent cesarean section during aortic surgery. The mean aortic cross-clamp time was 97.0(27.0) min and mean CPB time was 202.0(46.3) min. A total of 170 cases (90.9%) underwent DHCA or MHCA during the operations and the mean time was 22.0(13.0) min. 3 patients (1.6%) experienced the restart of CPB and the total operation time was 8.0(2.0) hours.

Postoperatively, 68 patients (36.4%) stayed in the ICU for > 7 days and 30 patients (16.0%) were intubated for > 5 days. A total of 11 patients (5.9%) received CRRT and 15 patients (8.0%) underwent a secondary surgery during hospitalization. 9 patients (4.8%) died during hospitalization.

**The basic characteristics of patients without acute aortic syndrome**

A total of 212 patients without AAS were enrolled in the non-AAS subgroup, amongst whom 212 patients (74.5%) were male, with a mean age of 57.0(14.8) years. The mean systolic blood pressure was 135.0(24.0) mmHg, with the mean diastolic blood pressure being 73.0(19.0) mmHg, and the mean MAP being 93.2(15.3) mmHg. 59.0% of patients were not smokers, and 62.7% of patients had no history of hypertension. Other comorbidities included diabetes (2.8%), peripheral vascular disease (7.5%), and chronic pulmonary disease (4.2%). 1 patient (0.5%) had recent myocardial infarction and 4 patients (1.9%) had a history of cardiovascular surgery. 14 patients (6.6%) had Marfan syndrome. 168 cases (79.2%) were diagnosed with ascending aortic dilatation or ascending aortic aneurysm, 38 patients (17.9%) with primary aortic root aneurysm, 4 patients (1.9%) with thoracic aortic aneurysm, 1 patient (0.5%) with the aneurysm of brachiocephalic trunk, and 1 patient (0.5%) with aortic arteritis. Preoperative laboratory examinations showed that the mean SCr was 70.9(20.1) μmol/L and the mean estimated eGFR was 98.4(17.3) ml/min/1.73m^2^. 9 cases (4.2%) had a preoperative SCr>1.2mg/dl. The mean LVEF value was 60.0%(7.5%).

The surgical options of patients without AAS were presented in supplementary table 3. During the surgery, a sum of 65 patients (30.7%) underwent the aortic arch surgeries, and 2 patients (0.9%) underwent the operations for descending aorta. In addition, 22 patients (10.4%) underwent CABG and 158 patients (74.5%) underwent valve replacement or plastic surgery. Mean aortic cross-clamp time was 83.0(29.5) min, and mean CPB time was 125.0(51.0) min. Only 23 patients (10.8%) received DHCA or MHCA. 2 patients (0.9%) experienced the restart of CPB and the total operation time was 6.0(1.5) hours.

Only 11 patients (5.2%) were treated in ICU for > 7 days, and 2 patients (0.9%) were intubated for > 5 days. Only 1 patient (0.5%) received CRRT, and 7 patients (3.3%) received a secondary surgery. 3 patients (1.4%) died during hospitalization.
