# Supplementary material for: Risk factors analysis of acute kidney injury following open thoracic aortic surgery in the patients with or without acute aortic syndrome: a retrospective study
Source: J Cardiothorac Surg. 2020 Aug 7;15:213. doi: 10.1186/s13019-020-01257-1 (PMC7412815; doi:10.1186/s13019-020-01257-1)
Supplement: Supplementary file 2 — Additional file 2. Supplementary Table 1 Surgical options of the overall cohort [file 13019_2020_1257_MOESM2_ESM.doc]

| **Supplementary Table 1 Surgical options of the overall cohort** | |
| --- | --- |
| Surgical options | n (%) |
| Sun's procedure | 160(40.1%) |
| Bentall procedure | 94(23.6%) |
| Ascending aorta and hemiarch replacement | 79(19.8%) |
| Ascending aorta replacement | 37(9.3%) |
| Modified Carbrol procedure | 17(4.3%) |
| Ascending aortic angioplasty | 4(1.0%) |
| Wheat procedure | 4(1.0%) |
| Total thoracic aortic replacement | 1(0.3%) |
| Thoracoabdominal aortic replacement | 1(0.3%) |
| Total aortic arch and thoracic aortic replacement | 1(0.3%) |
| David procedure | 1(0.3%) |
