# Supplementary material for: Risk factors analysis of acute kidney injury following open thoracic aortic surgery in the patients with or without acute aortic syndrome: a retrospective study
Source: J Cardiothorac Surg. 2020 Aug 7;15:213. doi: 10.1186/s13019-020-01257-1 (PMC7412815; doi:10.1186/s13019-020-01257-1)
Supplement: Supplementary file 3 — Additional file 3. Supplementary Table 2 Surgical options of AAS patient [file 13019_2020_1257_MOESM3_ESM.docx]

| **Supplementary Table 2 Surgical options of AAS patients** | |
| --- | --- |
| Surgical options | n (%) |
| Sun's procedure | 158(84.5%) |
| Ascending aorta and hemiarch replacement | 17(9.1%) |
| Bentall procedure | 6(3.2%) |
| Modified Carbrol procedure | 5(2.7%) |
| Ascending aorta replacement | 1(0.5%) |
