# Supplementary material for: Risk factors analysis of acute kidney injury following open thoracic aortic surgery in the patients with or without acute aortic syndrome: a retrospective study
Source: J Cardiothorac Surg. 2020 Aug 7;15:213. doi: 10.1186/s13019-020-01257-1 (PMC7412815; doi:10.1186/s13019-020-01257-1)
Supplement: Supplementary file 4 — Additional file 4. Supplementary Table 3 Surgical options of non-AAS patients [file 13019_2020_1257_MOESM4_ESM.docx]

| **Supplementary Table 3 Surgical options of non-AAS patients** | | |
| --- | --- | --- |
| Surgical options | n (%) | |
| Bentall procedure | 88(41.5%) | |
| Ascending aorta and hemiarch replacement | 62(29.2%) | |
| Ascending aorta replacement | 36(17%) | |
| Modified Carbrol procedure | 12(5.7%) | |
| Ascending aortic angioplasty | 4(1.9%) | |
| Wheat procedure | 4(1.9%) | |
| Sun's procedure | 2(0.9%) | |
| Total thoracic aortic replacement | 1(0.5%) | |
| Thoracoabdominal aortic replacement | 1(0.5%) | |
| Total aortic arch and thoracic aortic replacement | 1(0.5%) | |
| David procedure | 1(0.5%) |  |
